# Exploring the musical taste of expert listeners: musicology students reveal tendency toward omnivorous taste

**DOI:** 10.3389/fpsyg.2015.01252

**Published:** 2015-08-20

**Authors:** Paul Elvers, Diana Omigie, Wolfgang Fuhrmann, Timo Fischinger

**Affiliations:** ^1^Music Department, Max Planck Institute for Empirical AestheticsFrankfurt am Main, Germany; ^2^Institute of Musicology, University of ViennaVienna, Austria

**Keywords:** music, musical taste, musical preferences, expert listeners, personality, musical omnivorousness, familiarity, exposure

## Abstract

Musicology students are engaged with music on an academic level and usually have an extensive musical background. They have a considerable knowledge of music history and theory and listening to music may be regarded as one of their primary occupations. Taken together, these factors qualify them as ≫expert listeners≪, who may be expected to exhibit a specific profile of musical taste: interest in a broad range of musical styles combined with a greater appreciation of ≫sophisticated≪ styles. The current study examined the musical taste of musicology students as compared to a control student group. Participants (*n* = 1003) completed an online survey regarding the frequency with which they listened to 22 musical styles. A factor analysis revealed six underlying dimensions of musical taste. A hierarchical cluster analysis then grouped all participants, regardless of their status, according to their similarity on these dimensions. The employed exploratory approach was expected to reveal potential differences between musicology students and controls. A three-cluster solution was obtained. Comparisons of the clusters in terms of musical taste revealed differences in the listening frequency and variety of appreciated music styles: the first cluster (51% musicology students/27% controls) showed the greatest musical engagement across all dimensions although with a tendency toward ≫sophisticated≪ musical styles. The second cluster (36% musicology students/46% controls) exhibited an interest in ≫conventional≪ music, while the third cluster (13% musicology students/27% controls) showed a strong liking of rock music. The results provide some support for the notion of specific tendencies in the musical taste of musicology students and the contribution of familiarity and knowledge toward musical omnivorousness. Further differences between the clusters in terms of social, personality, and sociodemographic factors are discussed.

## Introduction

It has been noted since Antiquity that judgments on esthetic objects are difficult to account for. People who describe the physical properties of an artwork in similar ways will not necessarily end up with the same subjective evaluations of it. To account for these differences, the concept of ≫taste≪ – seen as a natural faculty or trait acquired by education or through socialization – was introduced into debates on esthetics. Differences in taste have been proposed to originate either from psychological differences such as humoral complexion and temperaments (Ficino, 1489/2002; Aﬄigemensis, 1950) or from sociological processes such as class distinction (Bourdieu, 1998). In any case, the possibility for members of society to acquire a refined taste through education and practice was seen as an important concern, and for centuries formed an integral part of pedagogical practices (Aristotle, 1984; Plato, 2006; Woerther, 2008). While musical taste and preference are often used synonymously, we treat them, in the current paper, as separate, albeit closely linked concepts. Both are concerned with the liking and disliking of music. However, ≫musical taste≪ describes more general evaluative attitudes while the term ≫musical preference≪ refers to direct evaluative judgments that are typically based on a comparison of two musical objects. Here, we consider the concept of taste to be the more relevant concept.

In the past decades, empirical findings on musical taste and preferences have corroborated the long existing notion that the music we like depends on a large variety of variables. Musical taste has been shown to be correlated with social and personal identity (North and Hargreaves, 1999; Abrams, 2009; Lonsdale and North, 2009), age, gender, and income (Behne, 1975; North, 2010; Bonneville-Roussy et al., 2013), big-five personality traits (Rawlings and Ciancarelli, 1997; Rentfrow and Gosling, 2003; Chamorro-Premuzic and Furnham, 2007; Delsing et al., 2008) and specific aspects of personality such as sensation-seeking or trait rebelliousness (Litle and Zuckerman, 1985; Carpentier et al., 2003).

The cultural and social environment influences the development of musical taste especially during adolescence when music making and listening become important activities (Behne, 1997; LeBlanc et al., 1996; North et al., 2000). The music that one listened to during adolescence is remembered better and is perceived as more emotionally arousing in later life stages (Schulkind et al., 1999; Krumhansl and Zupnick, 2013). Also, the musical taste formed during adolescence usually remains relatively stable across the life span (Holbrook and Schindler, 1989; Hemming, 2013).

The current study investigates the musical taste of musicology students, a *specific* group of adolescents and young adults. They generally have an extensive musical background and are more often than not skilled at playing at least one instrument. The musicologist’s main occupation is to listen to, understand, interpret, and analyze different forms of music, be they Western (classical, popular) or non-Western. Musicologists systematize and employ comparative techniques on music to ultimately gain an understanding of music history and theory across cultures and domains. Taken together, musicology students can be regarded as expert listeners who are preparing for a career involving intensive engagement with music. Their specialized education in music makes them a population of special interest and the question addressed by the current study is the extent to which these circumstances are manifested in a specific musical taste.

A great deal of research has examined musical experts with regard to their cognitive abilities (Sloboda, 1985, 2004), investigating for example how expertise relates to musical expectancies (Koelsch et al., 2002) or pitch processing (Besson et al., 2007). Another branch of research investigates the musical engagement and taste of people that are professionally involved in music such as musicians, musicologists, or music researchers. One of the first empirical works on this issue (Farnsworth, 1958) was a questionnaire study on the musical taste of an ≫American Elite≪, that was executed between 1938 and 1951 by members of the American Musicological Society (AMS). Members of the AMS were asked to indicate their favorite composers from a list of 474 names, resulting in two lists of favorite composers, one for those born since 1970, and one for eminent composers of all time. Notably the vast majority of the names on the list were western 18th and 19th century composers. L. v. Beethoven, J. S. Bach, and J. Brahms occupied the top three positions of all time eminent composers.

The relationship between musical training and a preference for musical styles considered as ≫serious≪ or ≫highbrow≪ has been reported in subsequent studies. Kelly (1961) found that people who study music instruments indicated a higher preference for classical music. Geringer and McManus (1979) identified a higher preference for traditional classical composers among music majors as compared to non-music majors. This relationship was replicated by Hargreaves et al. (1995), who found significant correlations between musical training and preferences for sophisticated musical styles such as classical, opera, and jazz. Gregory (1994) extended the scope of this research by showing that those who study music not only show a preference for classical music as compared to popular music but also an ≫instrumental bias≪ indicating that students tend to prefer music that is played on their own instrument. Ginocchio (2009) showed that musical training also leads to a greater appreciation of music in general regardless of the musical style and a similar pattern of taste has been found among professional music researchers (Wöllner et al., 2011). The latter study revealed a preference of classical music and ≫jazz/blues/RnB≪ over other musical styles. Additionally, the authors found a mean liking above the scale midpoint for all musical styles except rap/hip hop and dance/techno.

Taken together, the current literature indicates a greater liking by ≫expert listeners≪ of musical styles that are considered ≫highbrow≪ or ≫sophisticated≪. Additionally, Ginocchio (2009) and Wöllner et al. (2011) emphasize not only a greater appreciation of sophisticated styles but also a greater appreciation of music in general. This tendency can be described as the presence of an ≫omnivore≪-taste (Peterson and Kern, 1996) among musical experts. The term was originally introduced to describe a shift in musical taste among US-American ≫high-status persons≪ from liking only those considered as ≫elite≪ toward a greater liking of a variety of mid- and even low-brow musical styles. In contrast to features of a ≫snobbish≪ taste such as distinction and exclusion, omnivore taste has been linked to an openness of appreciation (Peterson and Kern, 1996; Warde et al., 2007) as well as openness to cultural diversity (Ollivier, 2008; Roose et al., 2012).

To extend previous findings on the influence of musical expertise, engagement, and familiarity on musical taste and to examine the notion of expert listeners as musical omnivores, we compared the musical taste of musicology students to that of a control group in a large-scale online survey. We enquired after their musical taste by soliciting details on the frequency with which they listened to 22 musical styles. Students with musicology as their major or minor subject were assigned to the group of ≫expert listeners≪, while other students were assigned to the control group. Bearing in mind that this criterion might be potentially biased – many non-musicology students also have an extensive background with music, and may be considered expert listeners – additional information on participants’ musical background, sociodemographic factors, and personality were obtained.

A factor analysis was used to reveal the underlying dimensions of musical taste. We then sorted participants based on a cluster analysis. Specifically, this method grouped participants according to their similarity in musical taste, regardless of their student status. We then examined the musical taste profiles of the clusters and the distribution of ≫expert listeners≪ and controls among them.

## Materials and Methods

### Ethics Statement

The study was conducted in full accordance with the *Ethical Guidelines* of the German Association of Psychologists (DGPs) and the *Ethical Principles of Psychologists and Code of Conduct* of the American Psychological Association (APA). These guidelines suggest that for the type of research reported here, a formal ethics approval is not necessary. This is due to the fact that the study only made use of completely anonymous questionnaires, i.e., no identifying information was obtained from the participants. Moreover participants were informed about the aim of the questionnaire, the anonymity of the data, and that participation was voluntary. In accordance with the ethical guidelines mentioned above it was not required to obtain informed consent.

### Participants

The sample (*n* = 1003) was made up of 647 (64.5%) students from the Humboldt University of Berlin, 98 (9.8%) students from the University of Vienna, 74 (7.4%) students from the Justus Liebig University Giessen, 20 (2%) students from the Catholic University Eichstätt-Ingolstadt, 2 (0.2%) pupils from the Albert Magnus Gymnasium, and 162 (16.1%) participants who did not indicate their university. The selection of universities was intended to control for possible regional biases and rather reflect the representative heterogeneity among students from different universities within Germany and Austria.

Of those who indicated their gender, 58% were female and 42% were male participants. The average age of participants was 24.13 and ranged from 17 to 66 years (SD = 5.08). 25% (*n* = 248) of the participants were musicology students, 64% (*n* = 639) reported other fields of study, and 11% (*n* = 116) made no indication of their subject. Those who did not indicate their subject of study were excluded from all group comparisons.

Participants were grouped as either expert listeners or controls based on their subject of study. Those who indicated musicology were grouped as ≫expert listener≪ while those who indicated other subjects belonged to the control group.

### Procedure

The data collection for the current study was part of a greater online survey about musical taste, engagement, and listening behavior among musicology and non-musicology students. The survey was conducted using an open source online service (https://www.soscisurvey.de/). Participants were recruited via mailing lists from the participating universities. The survey was open from the 22nd of January 2014 to the 12th of February 2014. Participants had the opportunity to participate in a lottery where they could win two concert vouchers to the amount of 25€.

### Measures

Musical taste was assessed by asking participants to indicate their frequency of listening to 22 music styles on five-point scales with endpoints ≫never≪ and ≫every day≪^1^. It was assumed that by assessing the frequency of listening rather than the reported liking of musical styles, one would provide a reliable measure reflecting actual listening behavior (Ter Bogt et al., 2011).

The musical styles were adapted from the revised version of the Short Test of Music Preferences (STOMP-R; Rentfrow and Gosling, 2003). Boer et al. (2013) pointed out that, since musical taste is culturally diverse, a cultural sensitive adaptation should improve the reliability of the measure. To this end, we excluded musical styles that were supposedly unfamiliar to German listeners and added styles that were not previously contained in the STOMP-R. Further, items that did not indicate a style but a musical function (religious) or a genre within a style (opera) were also excluded in order to generate a homogenous list with items mutually excluding each other. The only exception to this rule was the item soundtracks/theme songs. This category was retained because it has become a label of its own by which many people describe and sort their taste. The measure contained the following musical styles: rock, pop, classical, house, hip hop/rap, punk, soul/R&B, funk, jazz, oldies, heavy metal, blues, gospel, soundtracks/theme songs, country, alternative, folk, reggae, emo/screamo, dance/electronica, hard rock, and world music^2^.

A four-item-scale taken from the ≫16th Shell Youth Study≪ (Albert et al., 2010) was used to measure the social status of the participants. This standardized procedure asked participants for the school degree of their father, the monetary status of their family, the number of books present at home, and their housing situation.

Additional measurements regarding musical background and personality traits were taken in order to address expected individual differences in musical taste. Six items were employed to assess the musical background of participants. They measured the influence of different environments in which they actively engage with music on their musical development. On a four-point scale with endpoints at ≫often≪ and ≫never≪, participants were asked to indicate the frequency of their musical experiences within each of the following six typical environments for musical socialization: ≫kindergarten≪, ≫choir≪, ≫band≪, ≫ensemble≪, ≫solo-musician≪, ≫church≪. Personality traits were assessed using a five-item scale of the Big-Five-Inventory, which has previously been used and validated (Rammstedt et al., 2004). Although single-item measures of the big-five personality traits may not have the same quality as scales with multiple items for each dimension, the construct validity has been shown to be satisfactory (Rammstedt et al., 2004). As they only demand a little amount of time, they are especially useful in online surveys that make use of multiple scales.

### Analyses

To make full use of the richness of the data, a multivariate approach was chosen over bivariate group comparisons. We defined a twofold strategy: first, an exploratory factor analysis (Bachhaus et al., 2011) was employed to identify underlying dimensions of musical taste. Second, a hierarchical cluster analysis was performed on the basis of the dimensions of musical taste in order to identify potential differences between the groups.

The use of hierarchical cluster analysis allowed an unbiased analysis of potential heterogeneity among musicology students and controls. This method has proven successful in similar contexts where differences between two groups were expected and of interest but within-group homogeneity could not be assumed (Omigie et al., 2012). The hierarchical cluster analysis here was blind to the participants’ status as musicology student or not and simply grouped participants into clusters based on their similarity in musical taste. This approach accounts for a more diverse relationship between groups than traditional approaches and identifies potential subsets in the data. Since this is not the current standard procedure for the kind of research reported here, we have also provided results of traditional bivariate group comparisons of musicology students and controls in the supplementary material.

Exploratory factor analysis allowed the reduction of the variables to a few underlying dimensions. The use of this method in studies examining musical taste has grown in recent years, but there is mixed evidence in the literature regarding the *number* and *type* of dimensions that should be maintained. Rentfrow and Gosling (2003) found four dimensions underlying musical taste. However, in subsequent studies, this four-dimensional model was extended by a fifth dimension, resulting in the MUSIC-Model (Rentfrow et al., 2011, 2012), which was recently replicated (Bonneville-Roussy et al., 2013). Other studies have reported six underlying factors (e.g., Schafer and Sedlmeier, 2009).

Concerning the *type* of dimensions, there is no agreement as to whether they are related to specific musical characteristics or to musical styles and meta-styles. Rentfrow et al. (2011, 2012) interpreted the dimensions as being related to musical qualities as is clear in their use of the following labels: ≫Mellow≪, ≫Unpretentious≪, ≫Sophisticated≪, ≫Intense≪, and ≫Contemporary≪. Others (Schafer and Sedlmeier, 2009; Ter Bogt et al., 2011) seem to regard the dimensions as being more related to musical meta-styles. For instance Schafer and Sedlmeier (2009) proposed the following dimensions: ≫Sophisticated≪, ≫Electronic≪, ≫Rock≪, ≫Rap≪, ≫Pop≪, and ≫Beat, Folk and Country≪. To avoid having to make a subjective interpretation, we took the pragmatic approach of naming the underlying dimensions according to the music style, which had the highest loading on each dimension. For the sake of clarity, factor labels are written in bold typeface. This analysis was carried out using the principal() function from the ≫psych≪-package in the R environment (R Core Team, 2009).

Based on the identified dimensions, new variables were calculated for each participant by summing up each participant’s score for the musical styles belonging to one dimension and dividing this sum by the number of styles each dimension was comprised of. Next, these variables were passed on to a hierarchical cluster analysis (Everitt, 1974; Bachhaus et al., 2011), which grouped participants together based on their similarity in musical taste, regardless of their status as expert listener or control group. We used the hclust() function and specified the ≫ward≪ method, an agglomerative procedure based on a minimum-variance approach that starts with each participant as a single cluster and successively groups clusters that are most similar until all data are merged in one cluster. Since cluster analysis is a multivariate method, it allowed the inclusion of all six dimensions of musical taste in the analysis.

The resulting clusters revealed distinct profiles of musical taste, which finally served as a basis for the analysis of potential group differences in musical taste. It was assumed that, if in fact musicology and non-musicology students show distinct profiles in musical taste, we would obtain robust homogenous clusters, which separate them from each other.

For both types of analyses reported here, there are no standardized procedures for calculating statistical power or determining optimal sample size. However, a few rules of thumbs have been proposed: with regard to factor analysis MacCallum et al. (2001) recommended to use sample sizes not smaller than *N* = 500 and considered *N* > 1000 as excellent. The subject-to-variables (STVs) ratio should at least be 10:1 (Everitt, 1975). Concerning requirements of sample size for hierarchical cluster analysis, Formann (1984) recommended in a similar methodological context, a minimum sample size of *2^m^* where *m* is the amount of variables passed to the cluster analysis. For the cluster analysis of six musical taste dimensions this would require a minimum of 64 participants. With regard to the recommended procedures reported here our sample size is sufficient for subsequent analyses.

## Results

### Exploratory Factor Analysis

First, the optimal number of dimensions was determined by employing multiple methods: Kaiser-Guttman criterion (Guttman, 1954), scree plot (Cattell, 1966), and parallel analysis (Humphreys and Montanelli, 1975; Hayton et al., 2004). All methods suggested a six-factor solution as most appropriate for the data. The subsequently performed principal component analysis (PCA) with varimax rotation yielded six robust factors, which in total accounted for 60% of the variance (see **Table 1**).

**Table 1:** Principal component analysis with varimax rotation of 22 music styles across all participants.

	Six varimax rotated dimensions of musical taste					
Music styles	HARD ROCK	JAZZ	HOUSE	POP	FOLK	CLASSICAL
Hard rock	0.861					
Heavy metal	0.844					
Punk	0.666					
Rock	0.614					
Emo/screamo	0.588					
Jazz		0.782				
Blues		0.772				
Funk		0.759				
House			0.807			
Dance/electronica			0.800			
Hip hop			0.645			
Reggae			*0.443*			
Pop				0.792		
Soul/R&B				0.623		
Soundtracks				0.523		
Oldies				0.500		
Gospel				*0.471*		
Folk					0.834	
Alternative					0.641	
Country					0.565	
Classical						0.727
World music						*0.423*

*N = 1003. Primary factor loadings for 22 musical styles on six dimensions are displayed. Loadings below 0.50 are in italics.*

We further investigated whether the factor solution is due to the special population of musicology students in our sample. Therefore all methods for the determination of the factor structure were employed and subsequent PCAs for ≫non-musicology≪ and ≫only musicology≪ subsamples were computed. Factor structure heuristics yielded a five-factor solution for the non-musicology subsample, with a PCA accounting for 55% of the variance, compared to the only musicology subsample maintaining a six-factor solution and a PCA accounting for 63% of the variance, indicating that being a musicology-student only slightly affects the factor structure. Since our analyses were based on the comparison of musical taste among musicology students and controls, the PCA for the whole sample was retained. For the sake of completion, PCAs for subsets are included in the supplementary material.

### Obtained Dimensions

Styles on the underlying dimensions tended to share many musical characteristics. HARD ROCK encompassed musical styles generally characterized by the use of distorted electric guitars and loud, fast, noise-influenced sounds. In addition, all Hard Rock styles were historically related to each other with Hard Rock, Heavy Metal and Punk being derivatives of Rock and Emo/Screamo being a sub-style of Hardcore-Punk and related to Heavy Metal and Rock. The JAZZ dimension comprised of the three oldest and most important musical styles rooted in Afro-American music tradition. HOUSE encompassed mostly energetic musical styles that are closely related to dance and with a pronounced rhythmic contour. POP encompassed a heterogeneity of styles ranging from vocal-based to popular instrumental music. The FOLK dimension comprised of musical styles relating to the European-American music tradition, which typically involves singers accompanied by small ensembles of acoustic instruments. The dimension CLASSICAL encompassed ≫classical≪ western music and to a much lesser extent ≫world music≪, a term that is used to describe non-western art as well as traditional music from a western perspective.

### Hierarchical Cluster Analysis

**FIGURE 1:**
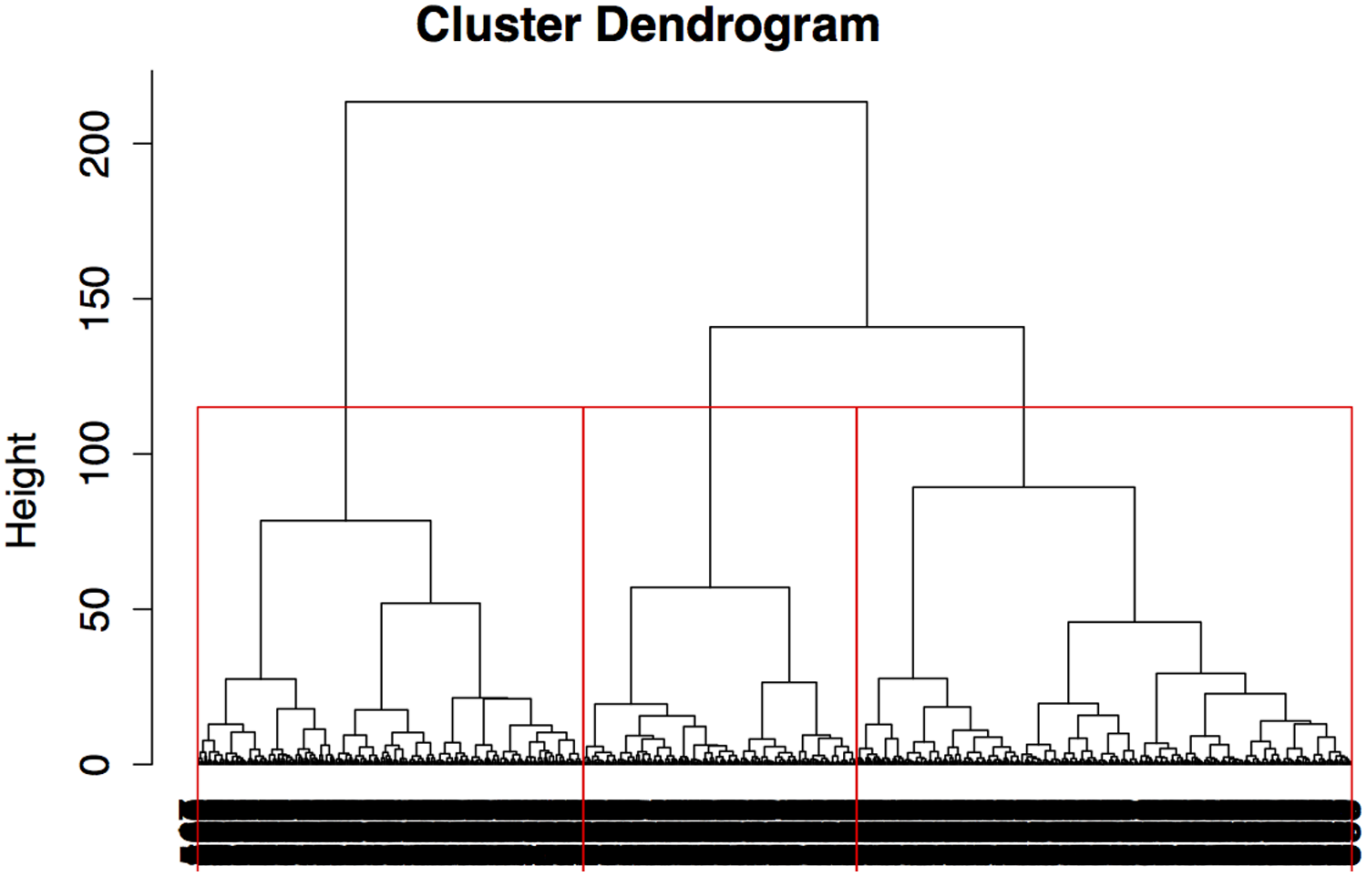
**Cluster-dendrogram of the hierarchical agglomeration showing a three-cluster-solution as indicated by the red lines.** Cluster description: 1 = Engaged Listeners, 2 = Rock Listeners, and 3 = Conventional Listeners.

The data set was transformed to a format suitable for the cluster analysis. It was decided to use a distance matrix with a Euclidean distance measure. **Figure 1** shows a cluster-dendrogram of the hierarchical agglomeration, which starts at the bottom with each case as a cluster until the top where all cases are merged into one cluster. The optimal number of clusters was determined by the maximal increase in cluster height, a criterion described by Everitt (1974). This resulted in a three-cluster-solution, as indicated by the red lines in the plot in **Figure 1**.

### Cluster-Profiles of Musical Taste

**FIGURE 2:**
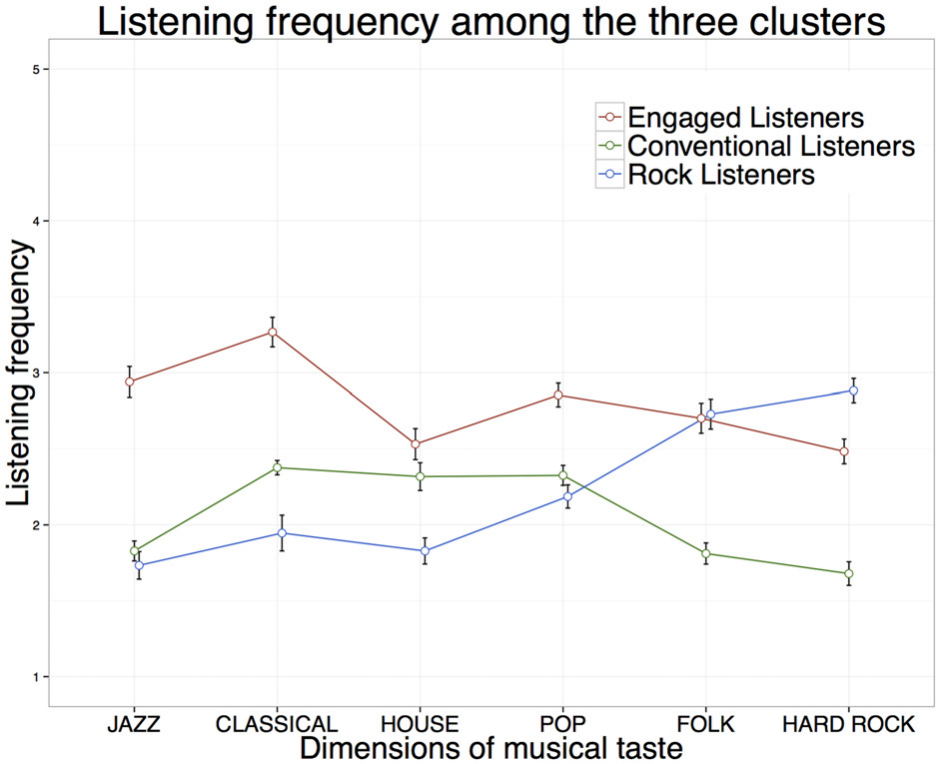
**Taste-profiles of identified clusters.** Means and 95% confidence intervals for listening frequency of ≫Engaged Listeners≪ (*n* = 285, red line), ≫Conventional Listeners≪ (*n* = 366 green line), and ≫Rock Listeners≪ (*n* = 202, blue line).

Following the clustering of participants into three groups that are, with respect to their musical taste, most homogenous, we analyzed their specific profiles of musical taste on the six dimensions. **Figure 2** displays the three profiles of musical taste indicating the mean frequency of listening for each of the six dimensions.

The profiles can be characterized in the following way: the first cluster showed the greatest musical engagement with four of the six dimensions and demonstrated peaks on JAZZ and CLASSICAL. This cluster was interpreted as ≫Engaged Listeners≪ (*n* = 285). The second cluster, which we labeled ≫Rock Listeners≪ (*n* = 202), showed a low engagement on the dimensions JAZZ, CLASSICAL, and HOUSE, but a great interest in the rock-related musical styles encompassed in the dimensions FOLK and HARD ROCK. The third cluster showed a medium engagement, in relation to the two other clusters, with a peak on the dimensions CLASSICAL, HOUSE, and POP, but the lowest values for FOLK and HARD ROCK. We labeled this cluster as ≫Conventional Listeners≪ (*n* = 366). All differences between the clusters on all dimensions were significant (*p* < 0.05) except those between ≫Conventional≪ and ≫Rock Listeners≪ on the dimension JAZZ as well as between ≫Engaged-≪ and ≫Rock Listeners≪ on the dimension FOLK (**Table 2**).

**Table 2:** Mean, SD, and one-way ANOVA of musical taste on six dimensions for the three clusters.

	ANOVA					Cluster mean and SD				
Musical dimensions	*df1*	*df2*	*F*	$\eta_{p}^{2}$	*p*	Engaged listeners	Conventional listeners	Rock listeners	Significance
JAZZ	2	850	238.3	0.36	<0.001	2.94 (0.86)	1.83 (0.64)	1.73 (0.65)	A,C
CLASSICAL	2	850	237.4	0.36	<0.001	3.70 (0.70)	2.38 (0.75)	1.94 (0.57)	A,B,C
HOUSE	2	850	43.6	0.09	<0.001	2.53 (0.87)	2.32 (0.89)	1.83 (0.62)	A,B,C
POP	2	850	83.11	0.16	<0.001	2.85 (0.66)	2.32 (0.64)	2.19 (0.55)	A,B,C
FOLK	2	850	153.2	0.26	<0.001	2.70 (0.84)	1.81 (0.68)	2.73 (0.71)	A,B
HARD ROCK	2	850	218.7	0.34	<0.001	2.48 (0.83)	1.68 (0.46)	2.88 (0.85)	A,B,C

*N = 853. Mean and SD in brackets. Significance for Bonferroni-corrected pairwise *t*-tests (*p* = 0.017). The capital letters indicate significant differences between group means where A = ≫Engaged Listeners≪ vs. ≫Conventional Listeners≪, B = ≫Conventional Listeners≪ vs. ≫Rock Listeners≪, and C = ≫Engaged Listeners≪ vs. ≫Rock Listeners≪.*

### Distribution of Musicology Students and Controls

**FIGURE 3:**
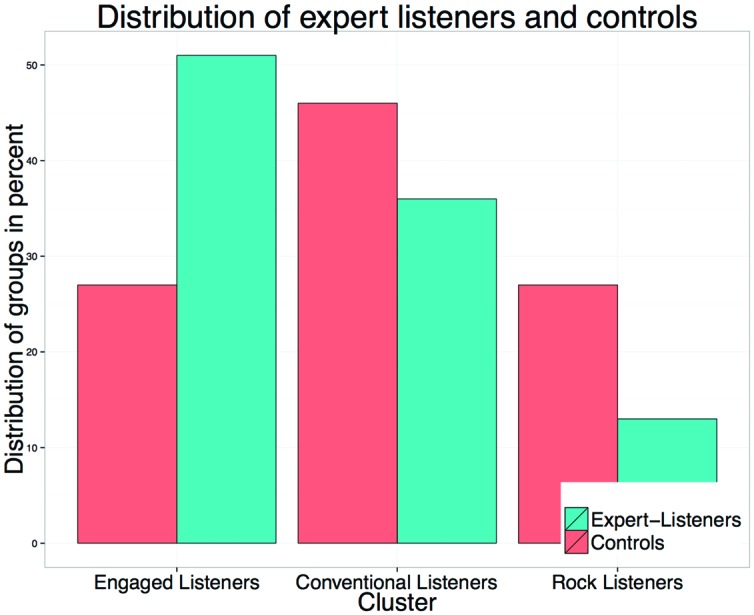
**Distribution of expert listeners and controls among the three clusters ≫Engaged Listeners≪, ≫Conventional Listeners≪, and ≫Rock Listeners≪ (in %)**.

Following the interpretation of the three clusters, the next step was to determine the proportion of expert listeners and controls among them. The analysis revealed clear trends among expert listeners and controls (**Figure 3**). More than half of the expert listeners (51%/*n* = 109) were included in the cluster of ≫Engaged listeners≪, while 36% (*n* = 77) were classified as ≫Conventional Listeners≪ and only 13% (*n* = 27) as ≫Rock Listeners≪.

On the other hand only approximately one third (27%/*n* = 129) of the control group was found in the cluster of ≫Engaged Listeners≪. Most of them (46%/*n* = 243) were ≫Conventional Listeners≪ and again approximately one third (27%/*n* = 147) of the controls were classified as ≫Rock Listeners≪. Chi-square tests for the groups of expert listeners and controls revealed that this distribution was not due to chance (χ^2^ = 44.379, *df* = 4, *p* < 0.001, Cramer’s *V* = 0.161). To sum up, expert listeners tended toward the cluster ≫Engaged Listeners≪ while the control group tended toward the cluster ≫Conventional listeners≪.

### Additional Variables

As a next step, additional external variables assessed within the survey were examined with respect to their potential of clarifying the distribution of participants in the three clusters.

### Musical Background

The musical background was assessed by the degree of influence of different musical environments that typically play a role in participant’s childhood and early adolescence. One-way ANOVAs for the types of musical background revealed clear differences between the clusters (**Table 3**).

**Table 3:** Mean, SD, and one-way ANOVA of musical background for the three clusters.

	ANOVA					Cluster mean and SD				
Musical environment	*df1*	*df2*	*F*	$\eta_{p}^{2}$	*p*	Engaged listeners	Conventional listeners	Rock listeners	Significance
Kindergarten	2	832	11.38	0.01	<0.005	2.65 (1.01)	2.41 (1.03)	2.42 (1.04)	A,C
Choir	2	810	48.69	0.03	<0.001	2.64 (1.35)	2.23 (1.33)	1.98 (1.28)	A,C
Band	2	818	82.41	0.06	<0.001	2.22 (1.38)	1.47 (1.01)	1.76 (1.21)	A,B,C
Ensemble	2	803	74.0	0.06	<0.001	2.19 (1.21)	1.73 (1.21)	1.41 (0.93)	A,B,C
Solo musician	2	840	49.48	0.05	<0.001	2.23 (1.18)	1.93 (1.02)	1.60 (0.85)	A,B,C
Church community	2	837	22.43	0.02	<0.001	2.03 (1.09)	1.73 (1.04)	1.59 (0.97)	A,C

*N = 853. Mean and SD in brackets. Significance for Bonferroni-corrected pairwise *t*-tests (*p* = 0.017). The capital letters indicate significant differences between group means where A = ≫Engaged Listeners≪ vs. ≫Conventional Listeners≪, B = ≫Conventional Listeners≪ vs. ≫Rock Listeners≪, and C = ≫Engaged Listeners≪ vs. ≫Rock Listeners≪.*

Additional pairwise comparisons with Bonferroni-corrected *t*-tests (*p* = 0.017) indicated that ≫Engaged Listeners≪ score significantly higher on all six items of the scale compared to ≫Conventional Listeners≪ as well as ≫Rock listeners≪. ≫Conventional Listeners≪ compared to ≫Rock Listeners≪ reported significantly greater influence of ≫ensemble≪, and significantly lesser influence of ≫bands≪ or as a ≫solo musician≪. Concerning ≫kindergarten≪, ≫choir≪ and ≫church community≪ the differences between ≫Conventional Listeners and ≫Rock Listeners≪ were not significant.

### Social Class

The employed measurement of social class grouped participants into five different levels of social status: ≫lower class≪, ≫lower middle class≪, ≫middle class≪, ≫upper middle class≪, and ≫upper class≪. Social status was distributed the following way in the whole sample (*n* = 1003): 6% (*n* = 36) of the participants were classified as ≫lower class≪, 28% (*n* = 239) as ≫lower middle class≪, 56% (*n* = 567) as ≫middle class≪, 10% (*n* = 107) as ≫upper middle class≪, and none as ≫upper class≪. Surprisingly, no significant differences between the three clusters were found with regard to social class, χ^2^ = 8.7638, *df* = 6, *p* = 0.18, Cramer’s *V* = 0.072.

### Age and Gender

There were no significant differences between the clusters with regard to age, *F*(2,783) = 0.13, *p* = 0.88. The distribution of gender within the three clusters showed a significant difference, χ^2^ = 11.097, *df* = 4, *p* = 0.025, Cramer’s *V* = 0.082, with 43% female participants among the ≫Engaged Listeners≪, 34% among the ≫Conventional Listeners≪, and 45% among the ≫Rock Listeners≪, as compared to 40% females among all participants.

### Personality

The analysis of group differences with regard to the big five personality traits revealed mixed results (**Table 4**). One-way ANOVAs for each of the big-five personality dimensions yielded significant differences only on three of five dimensions: extraversion, agreeableness, and conscientiousness. On the dimensions of emotional stability and openness the clusters did not differ significantly.

**Table 4:** Mean, SD, and one-way ANOVA of big-five personality dimensions for the three clusters.

	ANOVA					Cluster mean and SD				
Personality dimensions	*df1*	*df2*	*F*	$\eta_{p}^{2}$	*p*	Engaged listeners	Conventional listeners	Rock listeners	Significance
Extraversion	2	780	5.57	0.01	0.004	3.32 (1.62)	3.33 (1.51)	3.76 (1.63)	B,C
Emotional stability	2	780	0.06	0.00	0.945	3.85 (1.73)	3.89 (1.69)	3.89 (1.74)	NS
Openness	2	780	1.2	0.00	0.3	2.31 (1.38)	2.44 (1.29)	2.51 (1.20)	NS
Agreeableness	2	779	4.1	0.01	0.017	5.11 (1.55)	5.05 (1.47)	4.70 (1.54)	C
Conscientiousness	2	780	4.51	0.01	0.011	3.09 (1.58)	2.72 (1.43)	2.91 (1.51)	A

*N = 781. Mean and SDs in brackets. Significance for Bonferroni-corrected pairwise *t*-tests. The capital letters indicate significant differences between group means where A = ≫Engaged Listeners≪ vs. ≫Conventional Listeners≪, B = ≫Conventional Listeners≪ vs. ≫Rock Listeners≪, C = ≫Engaged Listeners≪ vs. ≫Rock Listeners≪, and NS = not significant.*

Subsequently performed Bonferroni-corrected pairwise *t*-tests (*p* = 0.017) revealed that the ≫Rock Listeners≪ scored significantly higher on extraversion than both other clusters, while the difference between ≫Engaged Listeners≪ and ≫Conventional Listeners≪ was only significant with regard to conscientiousness. On the dimension of agreeableness the only significant difference was between ≫Engaged Listeners≪ and ≫Rock Listeners≪. In sum, mixed evidence was found for the potential of big five personality traits to explain differences between the three clusters.

## Discussion

We identified six dimensions of musical taste, which served as a basis for a hierarchical cluster analysis, yielding three clusters of participants with distinct taste profiles. The clusters were interpreted as ≫Engaged Listeners≪, ≫Conventional Listeners≪, and ≫Rock Listeners≪. Musicology students and controls were unevenly distributed among the clusters, revealing a clear tendency for musicology students toward the ≫Engaged Listeners≪ cluster (whereby the majority of them grouped into this cluster). The profile of musical taste for this cluster showed the highest engagement on four of the six dimensions. ≫Engaged Listeners≪ exhibited the greatest engagement with the dimensions CLASSICAL and JAZZ, which in turn comprised of musical styles that have previously been conceptualized as ≫sophisticated≪ (Rentfrow and Gosling, 2003; Rentfrow et al., 2012; Bonneville-Roussy et al., 2013). The identified tendency in musical taste of musicology students can be described as ≫omnivorous≪ (Peterson and Kern, 1996) with an accentuated preference for CLASSICAL and JAZZ and converges with previous research on the musical taste of expert listeners (e.g., Hargreaves et al., 1995; Ginocchio, 2009; Wöllner et al., 2011).

The initial shift from ≫snob≪ to ≫omnivore≪ in musical taste described by Peterson and Kern (1996) was identified among ≫high-status≪ North-Americans. They claimed a tendency toward a broader musical interest in the higher social milieu, whereas so-called ≫lowbrow≪ musical taste remained style-specific. This linked the hypothesis of the musical omnivore to a specific social status. We suggest a larger role of musical expertise and development toward the cultivation of an omnivorous taste. Indeed our results show that most musicology students clustered as ≫Engaged Listeners≪. In addition, participants in this cluster reported the strongest influence of various environments on their musical development in childhood and early adolescence. Comparable results were found by Ter Bogt et al. (2011), who identified a type of ≫high-involved listeners≪ that exhibited both an omnivorous taste and the greatest overall engagement with music regarding average listening time. Taken together, the results support the notion that an omnivore musical taste and a greater overall engagement with music are positively correlated. Regarding participants’ social status no significant differences were found between the clusters. This means that omnivore musical taste does not necessarily entail a higher social status: a musical education and a high involvement with music can result in an omnivorous musical taste as well. Put differently, those who are generally more engaged in music and have a broader range of experiences with different musical styles also show a greater appreciation of different musical styles.

Omnivore taste has previously been linked to openness (Peterson and Kern, 1996; Warde et al., 2007) suggesting that an individual tendency to appreciate a wider range of musical styles is a driving factor of omnivore taste. However, our data revealed no significant differences regarding the personality trait ≫openness≪ between the three clusters. Our data therefore does not support a specific relationship between openness and omnivorous taste. ≫Engaged Listeners≪ only scored significantly higher on ≫conscientiousness≪ as compared to ≫Conventional Listeners≪ and significantly lower on ≫extraversion≪ as compared to ≫Rock Listeners≪. Also, although age has previously been shown to be an important predictor of musical taste (Holbrook and Schindler, 1989; Bonneville-Roussy et al., 2013) no significant differences between the clusters were found. This might be explained due to the homogenous age in our sample of mostly university students. Further, while sex is not usually considered a discriminating variable with regard to musical taste, female taste has previously been linked to mellow and soft musical styles, and male with harder styles such as rock (North et al., 2000). Our data revealed a different relationship between sex and musical taste with a higher representation of women in the clusters of ≫Engaged Listeners≪ and ≫Rock Listeners≪ as compared to ≫Conventional Listeners≪.

The omnivorous musical taste among musicology students might be explained by the fact that they ≫expose themselves≪ to various kinds of music and have spent a considerable amount of their time listening to music. On the level of single musical pieces it has been shown that exposure has a positive effect on appreciation and liking (Zajonc, 1968; Finnäs, 1989; Peretz and Gaudreau, 1998; Szpunar et al., 2004; North and Hargreaves, 2008). In addition to greater exposure, however, musicology students also engage with music on an academic level, allowing them to learn its rules. Learning the rules that underlie musical structure might be an important factor for having a deeper interest and greater appreciation of that musical style. Since these rules may vary considerably between different musical styles, those who have a greater and more diverse musical expertise may be more capable of experiencing different musical styles as rewarding. With regard to specific musical pieces it has been proposed that positive reward from music listening are linked to the familiarity with those pieces and may play a crucial part in the anticipation of musical events (Salimpoor et al., 2011).

## Conclusion

One may assume that the omnivorous musical taste of expert listeners results from a greater familiarity with a variety of musical styles and greater knowledge of a variety of rules that underlie musical structure. Since musicology students generally exhibit traits that have been treated as important factors influencing musical taste, they serve as an interesting population for research on musical taste and preferences. They are assumed to be familiar and knowledgeable with a variety of musical styles, which in turn is manifested in a greater involvement and appreciation of different kinds of music.

## Limitations and Future Research

We are aware that certain measurements that were employed in this study bore a limited potential for discrimination. For instance, the scale assessing participant’s musical background did not account for all potential environments that might have played a role in childhood. It will also be useful in future work to collect further data on musical activity such as hours of musical practice per week.

Musical taste was assessed by the frequency of listening to 22 musical styles. It was assumed that this measure would be easier to assess for participants to retrospectively access. However, it confounded the degree of liking with actual listening behavior. Even though these were assumed to be highly correlated, the assessment of each variable individually would reveal further information on musical taste.

Unfortunately, we cannot rule out any potential effects of demand characteristics or social desirability (Salganik et al., 2006) that might have played a role, particularly in the case of musicology students. While it was assumed that the kind of anonymous online survey reported here would keep demand characteristics low, it might have nevertheless been the case that the way musicologists reported on their musical taste was somewhat biased by what they thought was expected from them.

Finally, future research should employ measures of musical taste that are sensitive to more fine-grained differences. Since the aim of this study was to use a measure that has previously been validated and shown to be reliable, it was decided to use the modified version of the STOMP-R (Rentfrow and Gosling, 2003). However, it is possible that differences between musicology students and controls will become even more apparent with the use of an extended measure of musical taste.

## Conflict of Interest Statement

The authors declare that the research was conducted in the absence of any commercial or financial relationships that could be construed as a potential conflict of interest.

## Footnotes

1 In the original German version of the survey the endpoints were ≫nie≪ and ≫täglich≪.

2 The original German words for the musical styles were the following: Rock, Popmusik, Klassik, House/Techno/Rave, Hip Hop/Rap, Punk, Soul/R’n’B, Funk, Jazz, Oldies, Heavy Metal, Blues, Gospel, Soundtracks/Theme Songs, Country, Alternative, Folk, Reggae, Emo/Screamo, Dance/Electronica, Hardrock, Weltmusik.
