# Supplementary material for: Exploring the musical taste of expert listeners: musicology students reveal tendency toward omnivorous taste
Source: Front Psychol. 2015 Aug 20;6:1252. doi: 10.3389/fpsyg.2015.01252 (PMC4542684; doi:10.3389/fpsyg.2015.01252)
Supplement: Supplementary file 1 [file Table_1.DOCX]

***Supplementary Material***

**Exploring the musical taste of expert listeners: musicology students reveal tendency toward omnivorous taste**

**Paul Elvers*, Diana Omigie, Wolfgang Fuhrmann, & Timo Fischinger**

*** Correspondence:** [paul.elvers@aesthetics.mpg.de](mailto:paul.elvers@aesthetics.mpg.de)

1. **Bivariate group comparison of musicology students and controls**

Although an exploratory approach was preferred as the main analysis approach, for the sake of completeness, bivariate statistics of group differences between musicology and non-musicology students were computed. In order to see if there is a reliable difference between the musical taste of musicology and non-musicology students, a MANOVA with Group as the independent variable and the six dimensions of musical taste as dependent variables was carried out. The analysis revealed that differences are not due to chance *F*(6, 745) = 21.14, *p* < .001.

Subsequently performed t-tests for each dimension of musical taste showed that the two groups differ significantly on the dimensions JAZZ, HOUSE, and CLASSICAL but not on HARD ROCK, FOLK, and POP.

| **Supplementary Table 1.** *Means, SDs, and t-tests of Musical Taste on Six Dimensions for Expert Listeners and Controls* | | | | | | | |
| --- | --- | --- | --- | --- | --- | --- | --- |
|  | t-test | | | |  | Group Means & *SD*s | |
| Musical Dimensions | *df* | *t* | Cohen's *d* | *p* |  | Expert Listeners | Controls |
|  |  |  |  |  |  |  |  |
| JAZZ | 341.51 | 5.85 | 0.28 | <.001 |  | 2.52 (0.97) | 2.08 (0.83) |
| CLASSICAL | 428.46 | 9.20 | 0.45 | <.001 |  | 3.00 (0.75) | 2.42 (0.83) |
| HOUSE | 394.88 | -3.04 | -0.15 | .003 |  | 2.10 (0.97) | 2.31 (0.83) |
| POP | 387.29 | 0.82 | 0.04 | .4 |  | 2.52 (0.69) | 2.47 (0.67) |
| FOLK | 378.2 | -1.17 | -0.06 | .2 |  | 2.26 (0.88) | 2.35 (0.86) |
| HARD ROCK | 407.33 | -1.89 | -0.09 | .06 |  | 2.13 (0.83) | 2.26 (0.88) |
| Note. *N* = 853. | | | | | | | |

1. **PCAs for subsets of musicology students and non-musicology students.**

| **Supplementary Table 2.** *PCA for subset of non-musicology students* | | | | | |
| --- | --- | --- | --- | --- | --- |
|  | Five varimax rotated dimensions of musical taste | | | | |
| Music Styles | HARD ROCK | JAZZ | POP | HOUSE | FOLK |
| Hardrock | .831 |  |  |  |  |
| Heavy Metal | .790 |  |  |  |  |
| Rock | .709 |  |  |  |  |
| Punk | .707 |  |  |  |  |
| Alternative | .583 |  |  |  |  |
| Emo/Screamo | .566 |  |  |  |  |
| Jazz |  | .767 |  |  |  |
| Blues |  | .711 |  |  |  |
| Funk |  | .699 |  |  |  |
| Reggae |  | .509 |  |  |  |
| Gospel |  | .505 |  |  |  |
| World Music |  | *.412* |  |  |  |
| Pop |  |  | .795 |  |  |
| Soul/R&B |  |  | .591 |  |  |
| Country |  |  | .537 |  |  |
| Oldies |  |  | .515 |  |  |
| Soundtracks |  |  | *.497* |  |  |
| House |  |  |  | .765 |  |
| Dance/Electronica |  |  |  | .749 |  |
| Hip Hop |  |  |  | .660 |  |
| Folk |  |  |  |  | .734 |
| Classical |  |  |  |  | *.390* |
| *NOTE.* N = 639. Primary factor loadings for 22 musical styles on five dimensions are displayed. Loadings below .50 are in italics. | | | | | |

| **Supplementary Table 3.** *PCA for subset of musicology-students* | | | | | | |
| --- | --- | --- | --- | --- | --- | --- |
|  | Six varimax rotated dimensions of musical taste | | | | | |
|  | HARD ROCK | JAZZ | DANCE/  ELECTRO-NICA | SOUL/  RNB | FOLK | SOUNDTRACKS |
| Hard Rock | .883 |  |  |  |  |  |
| Heavy Metal | .867 |  |  |  |  |  |
| Punk | .695 |  |  |  |  |  |
| Rock | .629 |  |  |  |  |  |
| Jazz |  | .846 |  |  |  |  |
| Funk |  | .763 |  |  |  |  |
| Blues |  | .752 |  |  |  |  |
| World Music |  | *.424* |  |  |  |  |
| Dance/Electronica |  |  | .819 |  |  |  |
| House |  |  | .774 |  |  |  |
| Hip Hop |  |  | .670 |  |  |  |
| Reggae |  |  | .564 |  |  |  |
| Pop |  |  |  | .782 |  |  |
| Soul/R&B |  |  |  | .621 |  |  |
| Oldies |  |  |  | .531 |  |  |
| Gospel |  |  |  | *.444* |  |  |
| Folk |  |  |  |  | .797 |  |
| Alternative |  |  |  |  | .675 |  |
| Country |  |  |  |  | .641 |  |
| Soundtracks |  |  |  |  |  | .669 |
| Classical |  |  |  |  |  | *.491* |
| Emo/Screamo |  |  |  |  |  | *.487* |
| *NOTE.* N = 248. Primary factor loadings for 22 musical styles on six dimensions are displayed. Loadings below .50 are in italics. | | | | | | |
